# Improved functional properties of meat analogs by laccase catalyzed protein and pectin crosslinks

**DOI:** 10.1038/s41598-021-96058-4

**Published:** 2021-08-17

**Authors:** Kiyota Sakai, Yukihide Sato, Masamichi Okada, Shotaro Yamaguchi

**Affiliations:** Amano Enzyme Inc. Innovation Center, Kakamigahara, Japan

**Keywords:** Biochemistry, Biological techniques, Biotechnology, Microbiology

## Abstract

The gap between the current supply and future demand of meat has increased the need to produce plant-based meat analogs. Methylcellulose (MC) is used in most commercial products. Consumers and manufacturers require the development of other novel binding systems, as MC is not chemical-free. We aimed to develop a novel chemical-free binding system for meat analogs. First, we found that laccase (LC) synergistically crosslinks proteins and sugar beet pectin (SBP). To investigate the ability of these SBP-protein crosslinks, textured vegetable protein (TVP) was used. The presence of LC and SBP improved the moldability and binding ability of patties, regardless of the type, shape, and size of TVPs. The hardness of LC-treated patties with SBP reached 32.2 N, which was 1.7- and 7.9-fold higher than that of patties with MC and transglutaminase-treated patties. Additionally, the cooking loss and water/oil-holding capacity of LC-treated patties with SBP improved by up to 8.9–9.4% and 5.8–11.3%, compared with patties with MC. Moreover, after gastrointestinal digestion, free amino nitrogen released from LC-treated patties with SBP was 2.3-fold higher than that released from patties with MC. This is the first study to report protein-SBP crosslinks by LC as chemical-free novel binding systems for meat analogs.

## Introduction

In the last two decades, there has been a 58% growth in the global demand for meat due to an increase in the global population and rapid development of economy^1^. Moreover, the global population is expected to reach 9.7 billion by 2050, which will lead to a greater increase in protein demand^2^. However, limited land and water resources for livestock farming sustainability, rapid increase in animal welfare issues, undesirable effects on the environment, and change in climate have made it difficult to increase meat production to meet the future demand^3^. Thus, there is a gap between the present supply and future demand of meat; consequently, there is an increasing need to produce plant-based meat analogs as protein sources. Fresán and Sabaté reported that along with environmental benefits, human health benefits could also be achieved by changing the current dietary patterns to plant-based diets^4^. Moreover, to meet the demands of the expanding kosher and halal markets, it is necessary to develop plant-based meat analogs, instead of livestock-based or cell-based meat^5^. By 2025, more than 30% of the world’s population may consume these types of food^5^, making plant-based meat analogs one of the most popular topics within food and research communities.

Meat analogs are principally composed of textured vegetable proteins (TVPs) that imitate the fibrillar structure of meat muscle^6^. Soy-based TVP is a plant-based protein product that is cholesterol-free, with low concentrations of saturated fat and high concentrations of essential amino acids; it has several economical and functional benefits^7,8^. Soy-based TVP also presents characteristic functional properties, such as a high water holding capacity (WHC) and a good gelling behavior, fat absorption, and emulsification capacities^9^. It has been reported that when soy protein is used as a TVP constituent, the final product could mimic the texture, appearance, taste, smell, and functionality of red meat^5^.

The presence of binders is of importance as TVPs have no binding ability, considerably affecting the palatability of the final analog. Currently, methyl cellulose (MC) is used in most commercial products^10^ as it is a cheap binder and is considered safe for human consumption^11,12^. However, MC is chemically synthesized from cellulose and chloromethane in the presence of concentrated sodium hydroxide solution and is the main active ingredient in various laxatives. Therefore, concerns of MC not being a chemical-free binder and related risks necessitate the development of novel chemical-free binding systems for consumers and manufacturers. Moreover, the physical properties of existing plant-based meat analogs are still inferior to those of animal-based meat, especially when referring to texture, hardness, and juiciness^10,13^. These properties are crucial for the consumers’ acceptance of food products and, hence, remain a critical obstacle^13^. Another challenge for plant-based meat analogs is the presence of anti-nutritional factors, such as protease inhibitors, tannins, and phytates. These factors decrease the digestibility and bioavailability of plant proteins compared with animal proteins^10,13^. Based on the above issue, there are many remaining challenges in making plant-based meat analogs available to a wide range of consumers.

Protein crosslinks can be introduced into food matrices using chemical, enzymatic, and physical methods^14–16^. Among these approaches, transglutaminase (EC 2.3.2.13, TG) improves the physical properties of various protein-based foods^17,18^. This enzyme catalyzes the formation of an isopeptide bond between the glutamine residue side chains and lysine residue side chains^18^. Laccase (EC 1.10.3.2, LC) is also a protein-crosslink enzyme^19^. It oxidizes tyrosine phenols via a one-electron removal mechanism, producing phenoxy radicals. In proteins, the exposed tyrosyl side chains serve as substrates for oxidation by LC, resulting in the spontaneous production of subsequent protein crosslinks (dityrosine)^15,19–22^. However, the crosslinking reaction by LC oxidation cannot improve the physical properties of foods because proteins are poor substrates for LC^15,20,23,24^. Therefore, only TG has been commercially used as a crosslinking enzyme in the food industry.

Sugar beet pectin (SBP) consists of a linear α-1,4-d-galacturonic acid residue backbone, containing neutral sugars and ferulic acid, which are esters linked to arabinose and galactose side chains in the hairy region^25,26^. Approximately 50–60% of ferulic acid groups are linked to the backbone of the arabinan side chains, whereas the rest are linked to galactose residues^27^. Traditionally, SBP has been used in the food industry as an emulsifying, thickening, and stabilizing agent^26,28^; it also forms stable gels by oxidative coupling and covalent crosslinks between beet pectin molecules via oxidative enzymes, such as LC and peroxidase^29,30^. The presence of covalent crosslinks in gels prevents post-gelation structural rearrangement and associated syneresis. As the crosslinks introduced by LC are heat-stable, firmness and elasticity can be retained^29^. This hydrogel is thermo-irreversible^31^, which is a significant feature for food, biomedical, and biopharmaceutical applications^32^. So far, the use of an LC-catalyzed protein-SBP crosslink as a potential novel binding system for plant-based meat analogs has not been reported.

In this study, we aimed to develop a novel chemical-free binding system for meat analogs and to solve the physical and nutritional shortcomings of meat analogs. SBP was used as a mediator to improve the ability of LC to crosslink protein-based foods. First, we investigated whether TVP-SBP crosslinks formed by LC could become a novel binding system for meat analogs. As a result, the presence of LC and SBP improved the moldability and binding ability of patties, regardless of the type, shape, and size of TVPs. Moreover, compared with MC, LC-catalyzed protein-SBP crosslinks reduced the cooking loss and improved the water/oil holding capacity and gastrointestinal digestibility of meat analog patties. These findings indicate that TVP-SBP crosslinks formed by LC improved physical and nutritional properties, potentially solving the current challenges faced by plant-based meat analogs.

## Results and discussion

### Synergistic effect of LC on the formation of protein and SBP crosslinks

**Figure 1:**
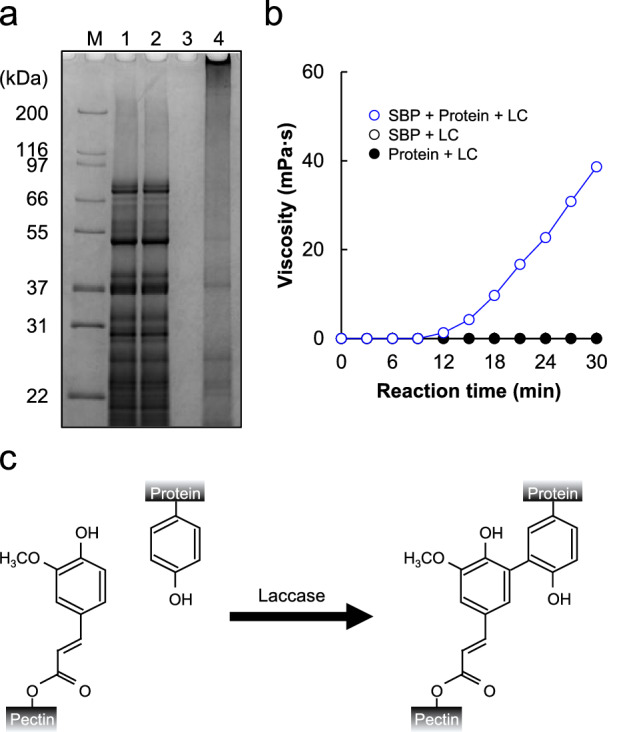
Synergistic effects of LC-catalyzed oxidative reaction on the formation of protein and SBP crosslinks. (**a**) SDS-PAGE analysis of soy protein and SBP treated with 100 U LC. Lane 1, non-treated soy protein and SBP; Lane 2, LC-treated soy protein; Lane 3, LC-treated SBP, Lane 4, LC-treated soy protein and SBP. Full-length SDS-PAGE gels are presented in Supplementary Fig. S1. (**b**) Apparent viscosity of soy protein and SBP solution treated with LC was measured using a viscometer. Open circle, LC-treated SBP; closed circle, LC-treated soy protein; blue circle, LC-treated soy protein and SBP. (**c**) Schematic of protein-SBP crosslink formation via LC-catalyzed oxidative reaction.

Soy protein solution, SBP solution, and soy protein + SBP solution were incubated with 100 units of LC for 20 min at 40 °C. As shown in lane 4, protein-SBP crosslinks were too large to enter the acrylamide gel (Fig. 1a). However, as shown in lanes 2 and 3, protein–protein crosslinks and SBP-SBP crosslinks were not induced by the LC reaction (Fig. 1a). The viscosity of the soy protein solution, SBP solution, and soy protein + SBP solution treated with LC was then measured. Although the viscosity of soy protein solution and SBP solution treated with LC did not change, the viscosity of soy + SBP solution treated with LC increased in a time-dependent manner (Fig. 1b). The soy + SBP solution treated with LC was finally gelled. These findings indicated that protein and SBP crosslinks were synergistically catalyzed by LC, leading to changes in their physical properties.

SBP gelation catalyzed by LC requires a longer incubation time and higher concentration of oxidation enzymes^29,33^. Previous studies have reported that proteins, such as fish gelatin, soy protein isolate, and whey protein isolate, can be crosslinked with SBP by oxidation enzymes^34–36^. As shown in Fig. 1c, LC can oxidize ferulic acid and tyrosine, leading to protein-SBP crosslinks^37,38^. This combination of SBP and protein provided additional properties to hydrogels and shortened the gelling time^29,33–39^. In this study, protein-SBP crosslinks were also formed by LC oxidation (Fig. 1a,b). Therefore, as a novel binding system for meat analogs, this protein-SBP gel could help develop food products with additional textural properties.

### Improving physical properties of meat analogs by SBP-protein crosslinks

**Figure 2:**
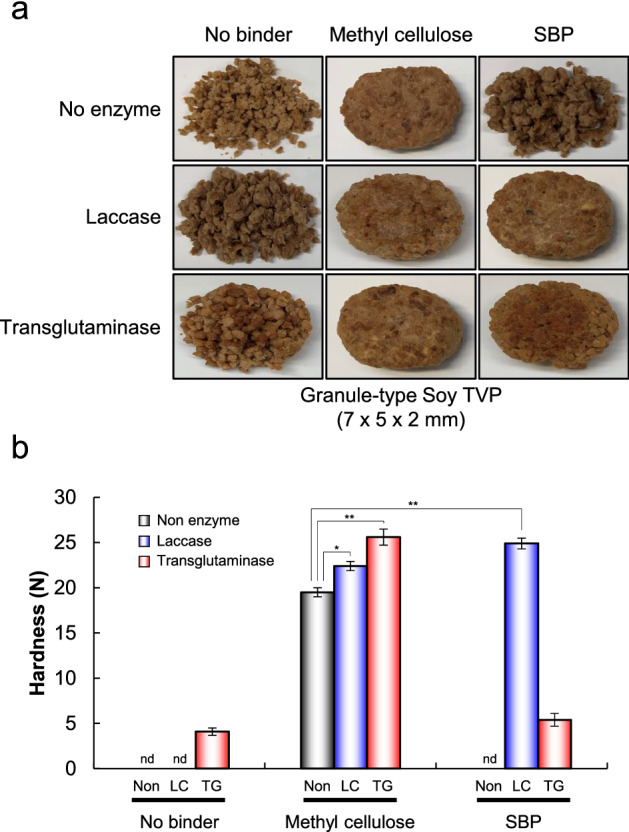
Appearance of grilled meat analogs treated with enzyme. (**a**) Meat analogs were treated with 20 U/g-TVP LC and 50 U/g-TVP TG, in the presence or absence of a binder (MC or SBP). (**b**) The hardness of meat analogs treated with each enzyme was measured using a rheometer. Data are presented as mean ± standard deviation of five experiments. *p < 0.05, **p < 0.01, Student’s *t*-test. Non, non-treated meat analogs; LC, LC-treated meat analogs; TG, TG-treated meat analogs.

To investigate whether SBP-protein crosslinks formed by LC oxidation could be a novel binder for meat analogs, soy-based TVP and SBP were incubated with LC. MC was used as the control. Figure 2a shows the meat analog patties after incubation and cooking. Non-treated and LC-treated meat analog patties without a binder collapsed after incubation and grilling. Meat analog patties containing MC maintained their shapes with or without enzymes after grilling. Interestingly, LC-treated meat analog patties containing SBP maintained their shape after grilling. In contrast, non-treated meat analog patties containing SBP collapsed after incubation and cooking. Moreover, various plant-based TVPs were incubated with SBP and LC under the same conditions (Supplementary Figs. S2 and S3). The results revealed that the presence of LC and SBP improved the moldability and binding ability of meat analog patties, regardless of the type, shape, and size of TVPs.

**Figure 3:**
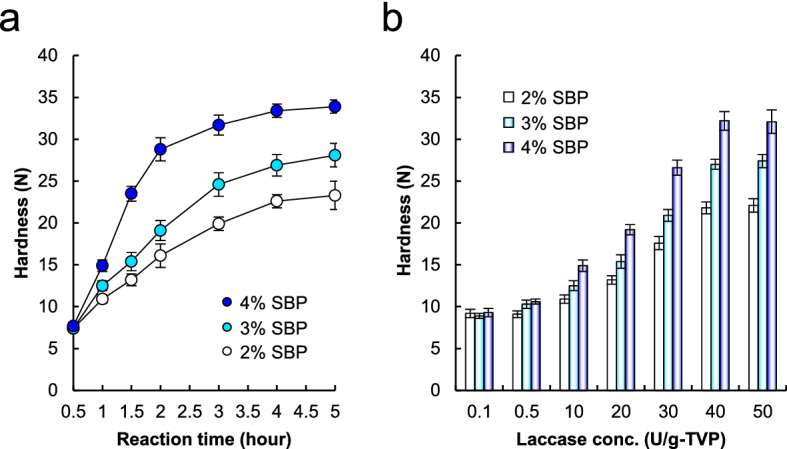
Hardness of grilled meat analog patties treated with LC. The hardness of the meat analogs treated with LC was measured using a rheometer. (**a**) Meat analogs containing 2–4% SBP were treated with 20 U/g-TVP LC at 0.5–4.0 h. (**b**) Meat analogs containing 2–4% SBP were treated with various concentrations of LC. Data are presented as mean ± standard deviation of five experiments.

The hardness of the meat analog patties after grilling was investigated using a rheometer (Fig. 2b). The hardness of patties containing MC was substantially higher in the order TG- > LC- > non-treated. Interestingly, the hardness of LC-treated patties containing SBP was substantially higher than that of non- and LC-treated patties containing MC. Moreover, as shown in Fig. 3, the hardness of meat analog patties was enhanced in an LC and SBP concentration- and time-dependent manner. The hardness of LC-treated patties with SBP plateaued at 32.2 N after 4 h of LC reaction, which was 1.7- and 7.9-fold higher than that of patties with MC and TG-treated patties. These findings indicate that TVP-SBP crosslinks offer great promise in changing the physical properties of plant-based meat analog products.

Table 1 shows the TPA parameters (hardness, cohesiveness, springiness, and chewiness) of enzyme-treated meat analog patties containing binders. The chewiness of these patties was substantially higher in the order LC-treated patties with SBP > non-treated and LC-treated patties with MC > TG-treated patties. The hardness and chewiness of LC-treated patties increased in a SBP concentration-dependent manner, whereas the cohesiveness and springiness did not change. Our finding that the hardness and chewiness of LC-treated patties increased in an SBP concentration-dependent manner is similar to that of previous studies that incorporated other binders into patties. These previous studies reported that increasing concentration of binders such as MC and carrageenan proportionally increases the hardness and chewiness of products^7,40–42^. The hardness of LC-treated patties containing SBP was lower than that of animal-based patties containing MC^40^. Meat protein generally presents a higher degree of shrinkage. Therefore, the higher hardness of animal-based patties has been suggested to be due to muscle protein denaturation, which led to meat hardening^40,43^. Whereas, the hardness and chewiness of LC-treated patties containing SBP were similar to or higher than those of meat analogs containing other binders reported previously^7,40,41,44^. These findings suggest that TVP-SBP crosslinks formed by LC oxidation are a novel binder for meat analog patties. This is the first report of enhancing the moldability and binding ability of patties by TVP-SBP crosslinks by LC.

**Table 1:** Texture profile analysis of meat analogs. Meat analogs were treated with 20 U/g-TVP LC or 50 U/g-TVP TG in the presence of MC or sugar beet pectin. Data are presented as mean ± standard deviation of five experiments.

Binder	Conc.	LC	TG	Hardness (N)	Cohesiveness	Springiness	Chewiness (N)
MC	2%	0 U/g-TVP	–	18.4 ± 0.6	0.70 ± 0.04	0.71 ± 0.05	9.1 ± 0.5
		20 U/g-TVP		21.5 ± 0.9	0.77 ± 0.03	0.81 ± 0.08	13.4 ± 0.7
SBP	2%	20 U/g-TVP	–	21.8 ± 0.3	0.78 ± 0.05	0.80 ± 0.05	13.6 ± 0.4
	3%			23.6 ± 0.5	0.77 ± 0.09	0.78 ± 0.07	14.2 ± 0.7
	4%			25.5 ± 0.7	0.79 ± 0.07	0.77 ± 0.06	15.6 ± 1.2
Non	–	–	50 U/g-TVP	7.1 ± 0.3	0.51 ± 0.06	0.57 ± 0.09	2.1 ± 0.3
MC	2%			23.1 ± 0.3	0.79 ± 0.08	0.77 ± 0.07	14.1 ± 0.7
SBP	2%			10.2 ± 0.4	0.57 ± 0.09	0.64 ± 0.08	3.7 ± 0.7

### Improving physical properties of meat analogs by MC

**Figure 4:**
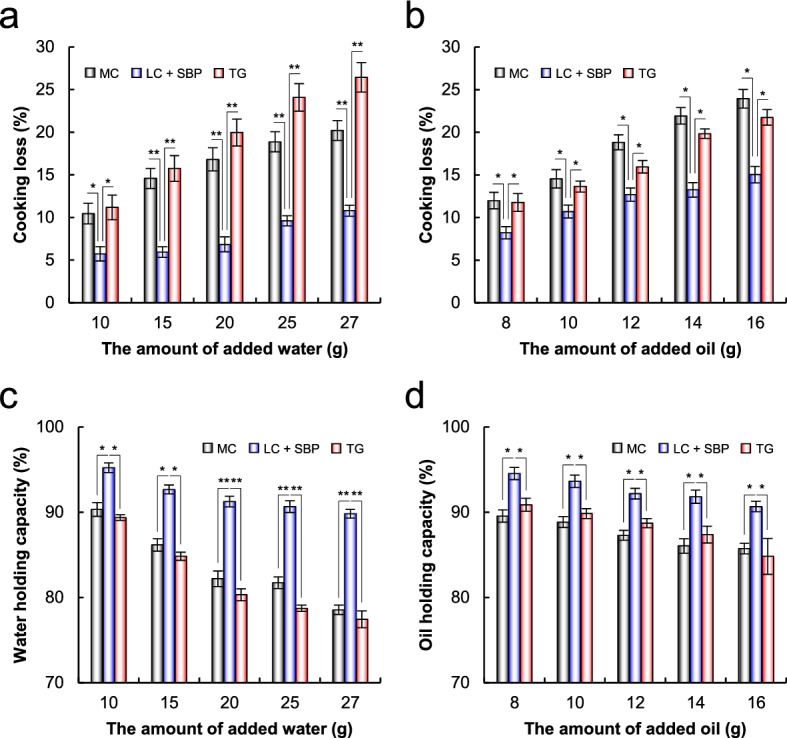
Cooking loss value and water/oil holding capacity of meat analogs. (**a**, **b**) Cooking loss was calculated as the percentage weight difference between the dough before cooking and after cooking. Meat analog containing different amounts of water (**a**) or oil (**b**) was treated with LC and cooked. (**c**, **d**) Holding capacity was calculated by comparing the weight of meat analogs before and after centrifugation. Meat analog patties containing different amounts of water (**c**) or oil (**d**) was treated with LC, cooked, and centrifuged. *p < 0.05, **p < 0.01, Student’s *t*-test. MC, meat analog containing MC; LC + SBP, LC-treated meat analog containing SBP; TG, TG-treated meat analog.

In this study, MC was used as the control in all experiments. The mechanism by which gelation between MC and protein is achieved remains unclear. One standard theory is that the hydrophobic methyl groups of MC are surrounded by cage-like structures of water molecules^45^. However, with increasing temperature, the cage structure is disrupted, and the polymers gradually lose water^46,47^. At elevated temperatures, MC prefers hydrophobic association states, leading to the formation of strong gels^46,47^; the results of this study are consistent with those reported previously (Figs. 2b, 4). After grilling, the cooking loss and holding capacity of patties decreased, indicating the release of water. In contrast, the hardness of patties that had MC increased. Moreover, all TPA parameters of the LC- and TG-treated patties containing MC were higher than those of non-treated patties containing MC (Table 1). These findings suggest that TPA parameters of the patty are synergistically enhanced by crosslinking enzymes such as LC and TG.

Currently, MC is used in most of the commercial products^10^. However, only a few studies have reported the effects of MC on the physical and functional characteristics of plant-based meat analog patties^40^. In this study, we investigated the properties of MC as a control binder, in detail. Selecting the right binder and its amount for meat analog products is crucial^10,40,48^. Therefore, the findings on patties containing MC expand our knowledge on their physical properties. This study will also contribute to a better understanding of the functional roles of MCs.

### Cooking loss and water/oil holding capacity of meat analog patties

Cooking loss represents the degree of meat shrinkage during cooking, which is an important indicator for evaluating the juiciness and yield of the final product. As the amount of water and oil increased, a typical increase in the cooking loss of all patties was observed (Fig. 4a,b). Surprisingly, the cooking loss of LC-treated patties containing SBP was substantially lower than that of patties containing MC and TG-treated patties. When adding various amounts of water and oil, the cooking loss of LC-treated patties with SBP decreased by up to 8.9–9.4% and 6.7–15.6% compared with patties with MC and TG-treated patties, respectively. Water and oil holding capacity is an important factor, as it affects the quality and yield of fresh patties or their products^49^; the higher the holding capacity of patties, the more the juiciness. As the amount of water and oil increased, the water and oil holding capacity of all patties showed a typical decrease (Fig. 4c,d). The water and oil holding capacity of LC-treated patties containing SBP was substantially higher than that of patties containing MC and TG-treated patties. When adding various amounts of water and oil, the water and oil holding capacity of LC-treated patties with SBP increased by up to 5.8–11.3% and 5.8–12.4% compared with patties with MC and TG-treated patties, respectively. These findings indicate that TVP-SBP crosslinks catalyzed by LC are superior to the high-network formed by MC, indicating that LC-treated patties containing SBP were juicier.

MC is an effective amphiphilic cellulose derivative with the ability to absorb high amounts of water and oil^40,50,51^. In this study, the cooking loss and holding capacity of patties were more enhanced by LC-catalyzed protein-SBP crosslinks than by the high-network formed by MC (Fig. 4). SBP also has good amphiphilic properties, contributed by the protein moiety ferulate and acetyl groups, which impart hydrophobic properties, and the carbohydrate fraction, which imparts hydrophilic properties^52,53^. Oxidative enzymes, such as LC and peroxidase, can form a hydrated network, leading to hydrogel formation with a high water-holding capacity^30^. Here, the water holding capacity of patties was in the order of SBP + LC > MC (Fig. 4a,c), suggesting that TVP-SBP crosslinks formed a hydrogel-like network and improved the water retention capacity of plant-based meat analog patties.

LC treatment is an effective approach to prepare stable multilayered emulsions as it can crosslink proteins and SBP, and shrink the droplet size^54,55^. A previous study reported that an LC-treated multilayered protein-SBP stabilized emulsion improves emulsifiability compared with a non-treated protein and SBP emulsion^56^. Moreover, emulsions treated with LC and SBP exhibit droplets with a tight protein-polysaccharide membrane, higher emulsification stability during heating, oil retention, and freeze–thaw cycles, and at a wide pH range^54–56^. The results of this study are consistent with those reported previously; the oil holding capacity of patties was in the order SBP + LC > SBP + TG > MC + TG > MC > TG (Fig. 4; Supplementary Fig. S4). It is considered that LC formed a multilayered protein-SBP-stabilized emulsion and enhanced the emulsion stability and oil retention capacity of plant-based meat analog patties.

### *In-vitro* gastrointestinal digestibility of meat analogs

**Figure 5:**
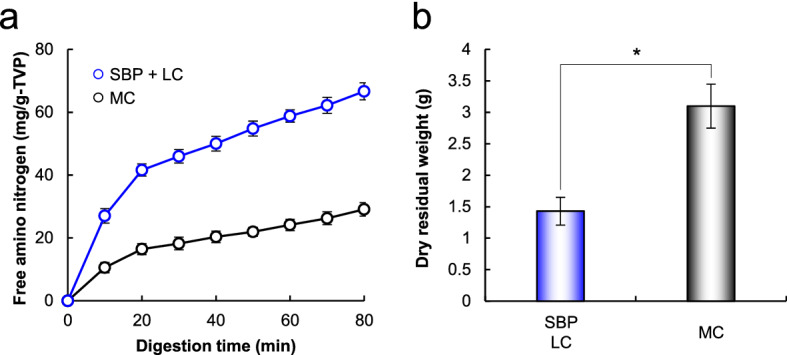
*In-vitro* gastrointestinal digestibility of meat analogs. The gastrointestinal digestion test was performed for 90 min. Free amino nitrogen released from meat analogs (**a**) was measured using the ninhydrin method, and then its dry residual weight (**b**) was measured. Data are presented as mean ± standard deviation of three experiments. *p < 0.05, Student’s *t-*test.

Figure 5 shows free amino nitrogen released from the plant-based meat analog patty and its dry residual weight after *in-vitro* gastrointestinal digestion by pepsin. The amount of free amino nitrogen released from LC-treated patties containing SBP was 2.3-fold higher than that from patties containing MC (Fig. 5a). The dry residual weight of LC-treated patties containing SBP was 2.2-fold lower than that of patties containing MC (Fig. 5b). These findings indicated that LC-treated patty containing SBP was easier to digest with pepsin and absorb nutrients from. This is considered to be because of higher water holding capacity, cohesiveness, and springiness (Fig. 4 and Table 1). Cohesiveness and springiness are the extent to which the gel withstands a second deformation relative to its resistance under the first deformation. Generally, the higher the water retention, cohesiveness, and springiness, the lower the density of food fragments after chewing. Therefore, the dispersibility of LC-treated patties containing SBP in the imitated gastric juice was considered to be high, leading to an enhanced pepsin activity toward the patties.

Meat as a protein source provides humans with the necessary nutrients and energy to function throughout the day^57^. Generally, meat analogs are considered to have slightly lower protein content than traditional meat products^10,13^. However, the digestibility of plant proteins is lower than that of animal-derived proteins because of different factors^58^. According to a previous study, all processing conditions, such as heat treatment, high pressure, pH change, protein fractionation, enzymatic reaction, milling, pressure, and fermentation, affect the nutritional availability of amino acids from proteins^59^. Here, replacing MC with TVP-SBP crosslinks catalyzed by LC as a binding system enhanced the digestibility of plant-based meat analog patties (Fig. 5a). Therefore, TVP-SBP crosslinks catalyzed by LC offer an option to overcome the poor nutritional availability of amino acids from plant-based meat analogs.

Based on recent scientific efforts, numerous studies have indicated the health benefits associated with the replacement of animal sources of protein with plant-based proteins, including reduced risks of type 2 diabetes, heart disease, and stroke^60,61^. Additionally, dietary fiber in meat analogs is considered to play an essential role in preventing large bowel disease, ischemic heart disease, and diabetes mellitus^62^. MC is a chemically modified polysaccharide, and this has raised concerns among the consumers and manufacturers as it is not a chemical-free binder. The SBP used in this study was a natural and chemical-free polysaccharide. Therefore, SBP offers great promises in not only enhancing physical and functional properties of patty but also improving nutritional availability and disease prevention.

## Materials and methods

### Materials

Granule-type, fillet-type, chunk-type, and slice-type soy-based TVPs were purchased from Marukome Co., Ltd. (Nagano, Japan). Granule-type, strip-type, and slice-type pea-based TVPs were obtained from Puris LLC (Minneapolis, USA), SANSHO Co., Ltd. (Tokyo, Japan), and AJIGEN Co., Ltd. (Kagawa, Japan), respectively. Slice-type gluten-based TVP was purchased from Saniku Foods Co., Ltd. (Chiba, Japan).

### SDS-PAGE analysis of crosslink

To investigate the synergistic effect of LC on the formation of SBP and protein crosslinks, the degree of crosslinking was measured by SDS-PAGE under reducing conditions with dithiothreitol (DTT). Assay of 10% (v/v) soy solution, 0.5% (v/v) SBP solution, and 10% (v/v) soy + 0.5% (v/v) SBP solutions was performed in reaction mixtures containing phosphate buffer (100 mM, pH 7.0) and LC (100 units). The reaction mixtures were incubated at 40 °C for 20 min, and the reaction was terminated by boiling at 100 °C for 5 min.

### Rheological characterization

The viscosity of the soy solution, SBP solution, and soy + SBP solutions was measured using EMS-1000 (Kyoto Electronics Manufacturing Co., Ltd., Tokyo, Japan). Each solution was assayed in reaction mixtures containing phosphate buffer (100 mM, pH 7.0) and LC (500 units). The shear rate applied to the samples was 200 s^−1^, and a preliminary measurement was conducted for 30 s to maintain the flow conditions. The temperature of the samples was set to 40 °C during the measurements. The variation in protein and SBP concentrations due to water vaporization was confirmed by gravimetric measurements.

### Preparation for plant-based meat analogs

The base of the TVP matrix was prepared using TVPs and a binder (MC or SBP), followed by the addition of olive oil and enzyme solution additives (Table S1). First, dried TVP was immersed in water (five times in volume) for 2 h for hydration. After dehydrating the swollen TVP, it was mixed 2.0–4.0% SBP or 2.0% MC at the final concentration. Unless otherwise noted, the TVP matrix was prepared in the absence and presence of 2.0% (final concentration) binder. Thereafter, different amounts of water or olive oil were added to each 25 g of TVP matrix. These were blended for 60 s using a hand blender. Thereafter, LC (LC-Y120; Amano Enzyme Inc., Nagoya, Japan) was added to the TVP matrix and blended for 60 s. LC used in this study was a commercially available food-grade product. According to the instruction manual of the manufacturer, the optimal reaction temperature for LC is 60 °C, with a preferred temperature range between 25 and 80 °C (> 65%). The TVP matrix was molded in a cylindrical mold (60 mm × 40 mm area and 25 mm height) and incubated at 25 °C for 4 h. The matrix was then cooked in an oven at 200 °C for 15 min and cooled to room temperature (20–25 °C) before being used for further analysis.

### Texture profile analysis

Texture profile analysis (TPA) was carried out using COMPAC-100II (Sun Scientific Co., Ltd., Tokyo, Japan) equipped with a cylindrical probe with an area of 31.4 mm. Meat analog patties were treated with 0.1–50 U/g-TVP LC and 50 U/g-TVP TG, respectively. Unless otherwise noted, it was performed with 20 U/g-TVP LC (5.0 mg). After grilling, meat analogs were prepared for the analysis and cut to a length of 15 mm to obtain homogeneous extrudates. The diameter of each extrudate was approximately 20 mm. A double compression cycle was performed at 1 mm/s until a recorded deformation of 50% was achieved. The following parameters were evaluated: hardness, the maximum force recorded during the first compression; cohesiveness, the area of work during the second compression divided by the area of work during the first compression; springiness, the distance recorded during the second compression divided by the distance of the first compression; and chewiness, hardness × cohesiveness × springiness.

### Cooking loss

The cooking method and conditions were determined based on the study of Pathare and Roskully^63^. The patties were cooked at 200 °C for 15 min, depending on the temperature at the center of the meat analog, reaching 80 °C. After cooking, the samples were cooled to room temperature (20–25 °C). Cooking loss was calculated as the percentage weight difference between the patty before cooking and after cooking, using the following formula: cooking loss (%) = ((W_1_ − W_2_)/W_1_) × 100; W_1_: weight of the meat analog before grilling (g), W_2_: weight of the meat analog after grilling (g).

### Water/oil holding capacity

Water holding capacity and oil holding capacity were measured by modifying a previously described method^64,65^. Briefly, grilled meat analog (5 g) was placed in a 50-mL tube with gauze underneath. The tube was then centrifuged at 3,000 g for 10 min at 35 °C. Holding capacity (HC) was calculated by comparing the weight of the meat analogs before and after centrifugation using the following formula: HC (%) = (W_2_/W_1_) × 100; W_1_: weight of the meat analog before centrifugation (*g*), W_2_: weight of the meat analog after centrifugation (*g*).

### *In-vitro* gastrointestinal digestibility of meat analogs

*In-vitro* gastrointestinal digestion tests were performed in 130-mL reaction mixtures containing NaCl (4.39 g), KCl (0.22 g), CaCl_2_ (0.04 g), McIlvaine buffer (pH 5.0), meat analogs (25 g, finely cut), and 0.0065% pepsin from porcine gastric mucosa (Sigma-Aldrich, St. Louis, MO, USA). The digestive reaction mixtures were incubated at 37 °C and 60 rpm for 90 min, and 1 N HCl was added to the reaction mixtures every 5 min, resulting in pH 3.0 after 40 min. The solution was clarified using a Nanosep® Centrifugal Device (Pall Corporation, Port Washington, NY, USA) as described in the instruction manual. The free amino nitrogen was measured using the ninhydrin method^66^.

## Conclusions

In this study, the synergistic effects of TVP and SBP crosslinks catalyzed by LC, on the physical and nutritional properties of meat analogs were investigated. The presence of LC and SBP improved the moldability and binding ability of meat analog patties, regardless of the type, shape, and size of TVPs. The hardness of the LC-treated patties plateaued at 32.2 N, indicating that TVP-SBP crosslinks formed by LC were superior to those formed by MC. In addition, the water/oil holding capacity of LC-treated patties containing SBP was substantially higher than that of patties containing MC. This indicates that LC-treated patties containing SBP were juicier. Moreover, the amount of free amino nitrogen released from LC-treated patties containing SBP was 2.3-fold higher than that from patties containing MC after *in vitro* gastrointestinal digestion. It is considered that LC-treated patties containing SBP are easier to digest using pepsin and absorb nutrients from. This study is the first to show that this protein-SBP crosslink catalyzed by LC could be a binding system for plant-based meat analogs.

## Supplementary Information

Supplementary Information.

## Data Availability

All data generated or analyzed during this study are included in this published article and supplementary information.
