# Supplementary material for: Improved functional properties of meat analogs by laccase catalyzed protein and pectin crosslinks
Source: Sci Rep. 2021 Aug 17;11:16631. doi: 10.1038/s41598-021-96058-4 (PMC8370993; doi:10.1038/s41598-021-96058-4)
Supplement: Supplementary file 1 — Supplementary Information. [file 41598_2021_96058_MOESM1_ESM.docx]

*Supplementary Files*

**Improved Functional Properties of Meat Analogs by Laccase Catalyzed Protein and Pectin Crosslinks**

**Kiyota Sakai^*^, Yukihide Sato, Masamichi Okada and Shotaro Yamaguchi**

**
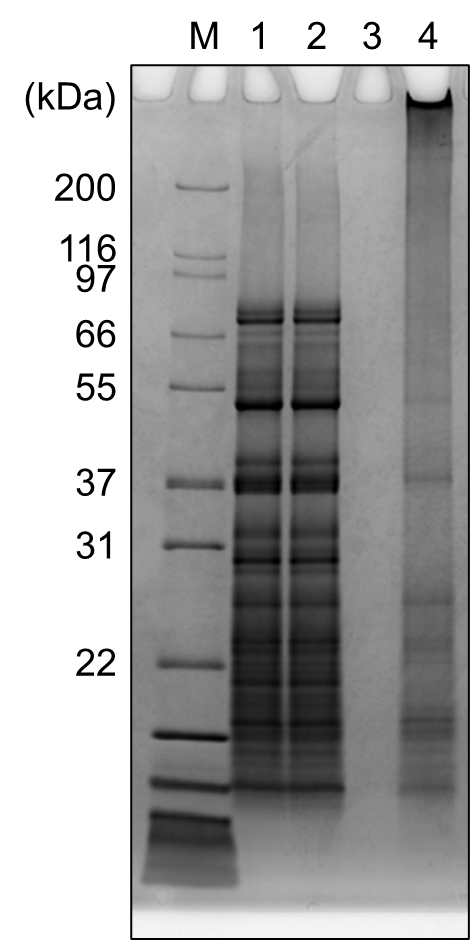
**

**Figure S1. SDS-PAGE analysis of soy protein and SBP treated with 100 U LC.**

Supplementary Figure 1 corresponds to Figure 1a.

**
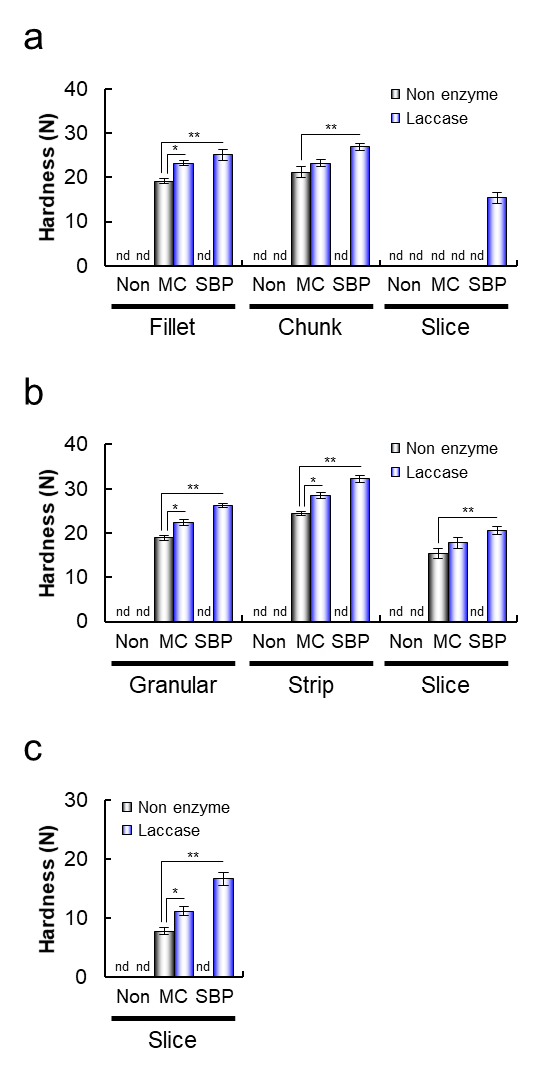
**

**Figure S2. Hardness of meat analogs prepared from various TVPs.**

Various types of TVPs (plant origin, shape, and particle size) were investigated. Meat analogs were prepared from soy-based TVP (a), pea-based TVP (b), and gluten-based TVP (c). Non-meat analogs containing no binder; MC, meat analogs containing MC; SBP, meat analogs containing SBP. Black bar, non-treated meat analogs; blue bar, LC-treated meat analogs.


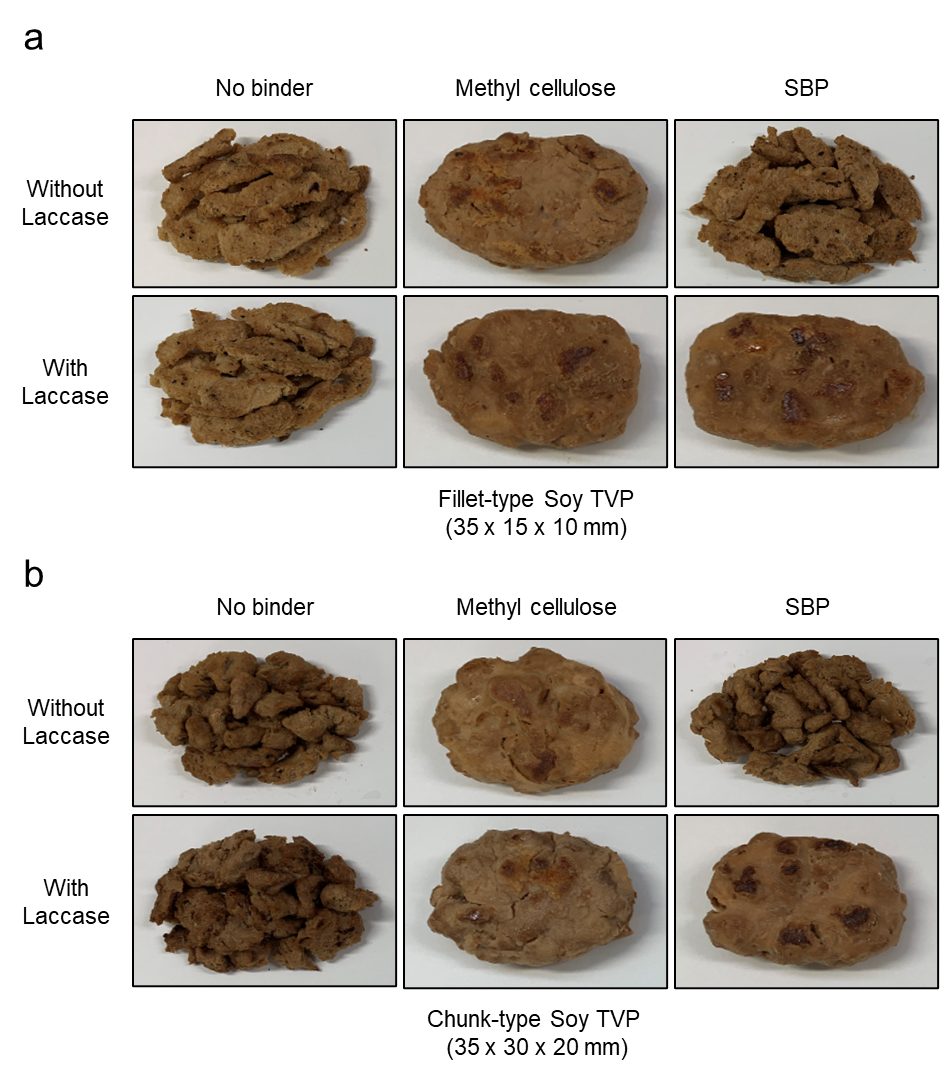


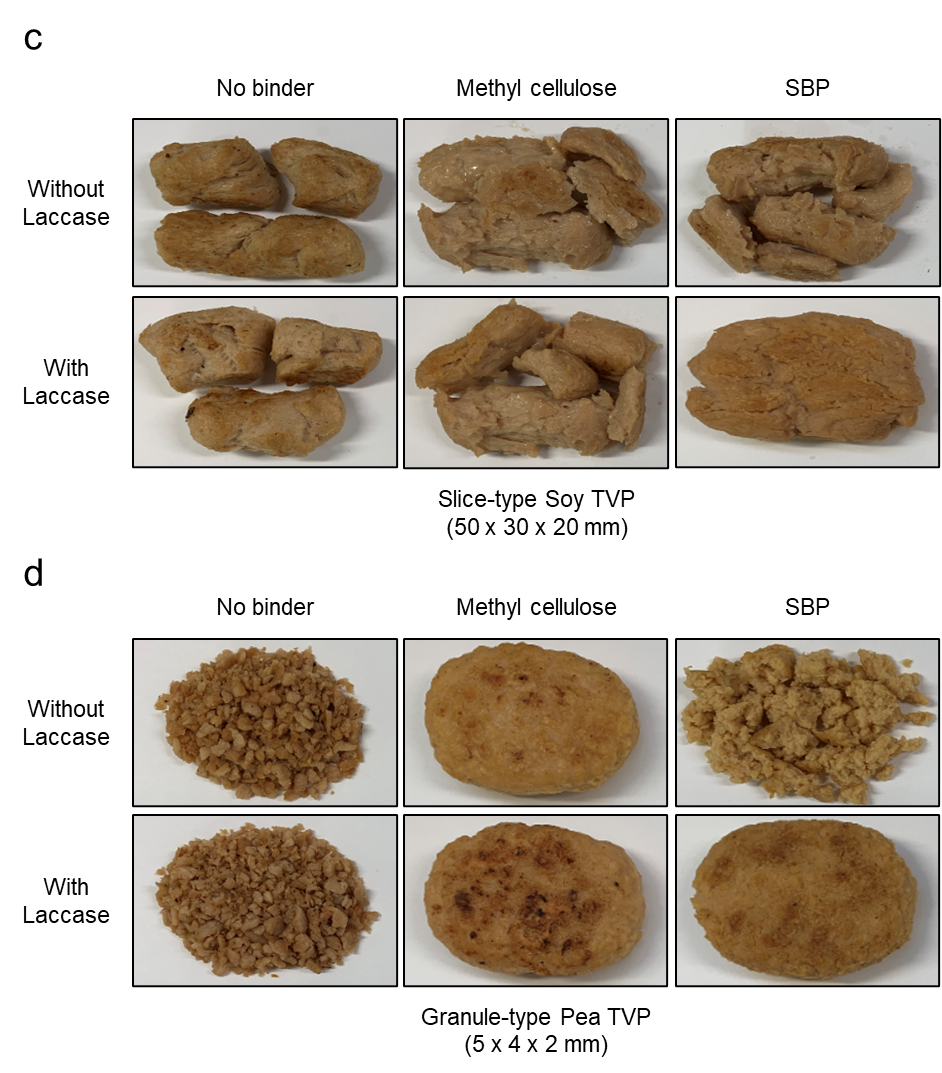


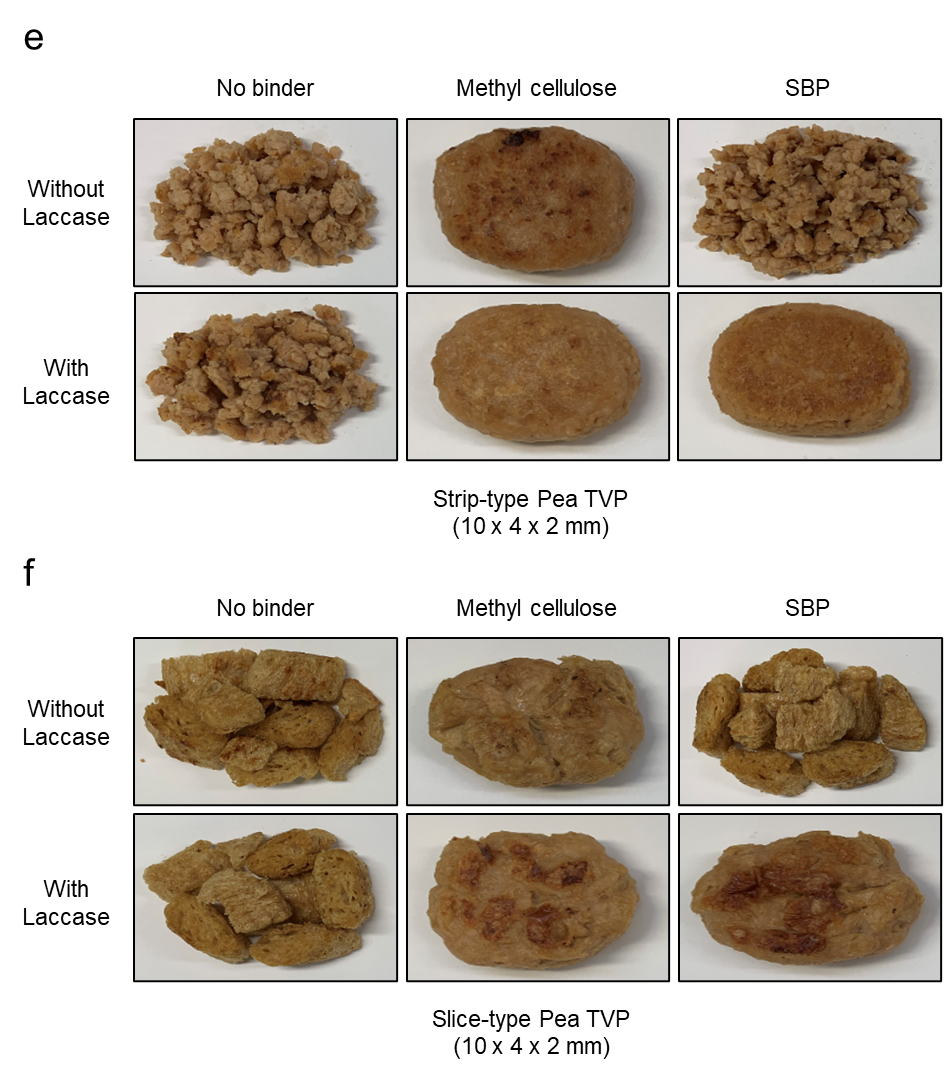


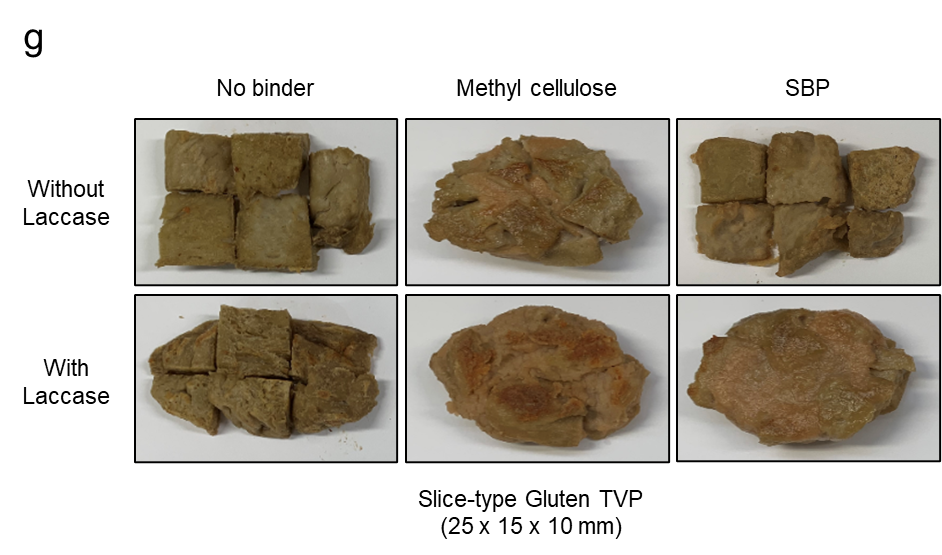


**Figure S3. Appearance of grilled meat analogs treated with enzyme.**

Various types of TVPs (plant origin, shape, and particle size) were investigated. (a) fillet-type soy-based TVP (35 × 15 × 10 mm); (b) chunk-type soy-based TVP (35 × 30 × 20 mm); (c) slice-type soy-based TVP (50 × 30 × 20 mm); (d) granule type pea-based TVP (5 × 4 × 2 mm); (e) strip-type pea-based TVP (10 × 4 × 2 mm); (f) slice type pea-based TVP (10 × 4 × 2 mm); (g) slice type gluten-based TVP (25 × 15 × 10 mm).


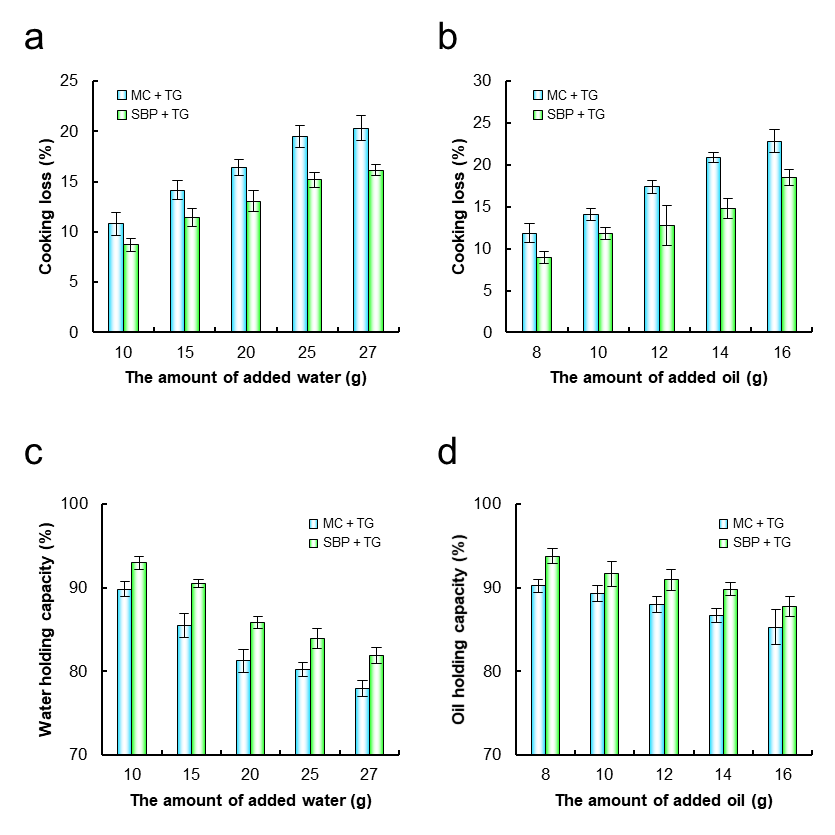


**Figure S4. Cooking loss value and water/oil holding capacity of meat analogs treated with TG.**

(a, b) Cooking loss was calculated as the percentage weight difference between the dough before cooking and after cooking. Meat analog with different amounts of water (a) or oil (b) was treated with TG and cooked. (c, d) Holding capacity was calculated by comparing the weight of meat analogs before and after centrifugation. Meat analog with different amounts of water (c) or oil (d) was treated with TG, cooked, and centrifuged. **p* < 0.05, Student’s *t*-test. MC + TG, TG-treated meat analog including MC; SBP + TG, TG-treated meat analog including SBP.

**Table S1. Mixing amounts of additives or enzymes in plant-based meat analog patties.**

|  | Wet TVP (g) | Water^1^ (g) | Oil^1^ (g) | MC (%) | SBP (%) | LC (U/g-TVP) | TG (U/g-TVP) |
| --- | --- | --- | --- | --- | --- | --- | --- |
| Non-treated patties without binder | 25 | 5 | 8 | - | - | - | - |
| Non-treated patties with MC | 25 | 5 | 8 | 2 | - | - | - |
| Non-treated patties with SBP | 25 | 5 | 8 | - | 2-4 | - | - |
| LC-treated patties without binder | 25 | 5 | 8 | - | - | 20 | - |
| LC-treated patties with MC | 25 | 5 | 8 | 2 | - | 20 | - |
| LC-treated patties with SBP | 25 | 5 | 8 | - | 2-4 | 20 | - |
| TG-treated patties without binder | 25 | 5 | 8 | - | - | - | 50 |
| TG-treated patties with MC | 25 | 5 | 8 | 2 | - | - | 50 |
| TG-treated patties with SBP | 25 | 5 | 8 | - | 2-4 | - | 50 |

^1^ In the cooking loss and holding capacity test, the amounts of water and oil ware 10–20 g and 8–16 g, respectively.
